# A Developmental Engineering-Based Approach to Bone Repair: Endochondral Priming Enhances Vascularization and New Bone Formation in a Critical Size Defect

**DOI:** 10.3389/fbioe.2020.00230

**Published:** 2020-03-31

**Authors:** Fiona E. Freeman, Meadhbh Á. Brennan, David C. Browe, Audrey Renaud, Julien De Lima, Daniel J. Kelly, Laoise M. McNamara, Pierre Layrolle

**Affiliations:** ^1^Trinity Centre for Biomedical Engineering, Trinity Biomedical Sciences Institute, Trinity College Dublin, Dublin, Ireland; ^2^Department of Mechanical and Manufacturing Engineering, School of Engineering, Trinity College Dublin, Dublin, Ireland; ^3^Biomechanics Research Centre (BMEC), Biomedical Engineering, National University of Ireland Galway, Galway, Ireland; ^4^INSERM, UMR 1238, PHY-OS, Laboratory of Bone Sarcomas and Remodelling of Calcified Tissues, Faculty of Medicine, University of Nantes, Nantes, France; ^5^Department of Anatomy, Royal College of Surgeons in Ireland, Dublin, Ireland; ^6^Advanced Materials and Bioengineering Research Centre (AMBER), Royal College of Surgeons in Ireland and Trinity College Dublin, Dublin, Ireland

**Keywords:** endochondral ossification, intramembranous ossification, bone tissue engineering, pre-vascularization, mesenchymal stem cells

## Abstract

There is a distinct clinical need for new therapies that provide an effective treatment for large bone defect repair. Herein we describe a developmental approach, whereby constructs are primed to mimic certain aspects of bone formation that occur during embryogenesis. Specifically, we directly compared the bone healing potential of unprimed, intramembranous, and endochondral primed MSC-laden polycaprolactone (PCL) scaffolds. To generate intramembranous constructs, MSC-seeded PCL scaffolds were exposed to osteogenic growth factors, while endochondral constructs were exposed to chondrogenic growth factors to generate a cartilage template. Eight weeks after implantation into a cranial critical sized defect in mice, there were significantly more vessels present throughout defects treated with endochondral constructs compared to intramembranous constructs. Furthermore, 33 and 50% of the animals treated with the intramembranous and endochondral constructs respectively, had full bone union along the sagittal suture line, with significantly higher levels of bone healing than the unprimed group. Having demonstrated the potential of endochondral priming but recognizing that only 50% of animals completely healed after 8 weeks, we next sought to examine if we could further accelerate the bone healing capacity of the constructs by pre-vascularizing them *in vitro* prior to implantation. The addition of endothelial cells alone significantly reduced the healing capacity of the constructs. The addition of a co-culture of endothelial cells and MSCs had no benefit to either the vascularization or mineralization potential of the scaffolds. Together, these results demonstrate that endochondral priming alone is enough to induce vascularization and subsequent mineralization in a critical-size defect.

## Introduction

Successful reconstruction of large bone defects remains an important challenge for reconstructive surgeons. Autologous bone grafting, using bone harvested from the patient’s own body, remains the gold standard for healing large bone defects, albeit that significant donor site morbidity has been reported and the quantity of bone available for grafting is limited (Reid, 1968; Coventry and Tapper, 1972; Younger and Chapman, 1989; Ahlmann et al., 2002; Finkemeier, 2002; St John et al., 2003; Brydone et al., 2010; Dimitriou et al., 2011). Recent studies have proposed that *in vitro* tissue engineering approaches should strive to simulate *in vivo* developmental processes and thereby imitate natural factors governing cell differentiation and matrix production, following the paradigm defined as “developmental engineering” (Lenas et al., 2009). During early fetal development, bone is formed via two specific mechanisms: intramembranous ossification and endochondral ossification. Both mechanisms begin with a two-step process whereby an organic matrix (osteoid/cartilage template) is initially laid down by osteoblasts/chondrocytes and then mineral crystals are produced and grow slowly over time to produce bone tissue (McNamara, 2011). These processes are distinguished from each other by the fact that the intramembranous process does not rely on the production of a cartilage template but the mesenchymal stromal cells (MSCs) form bone directly, whereas endochondral ossification involves the formation and remodeling of a cartilaginous template together with its vascularization.

Tissue engineering approaches have typically focused on the direct osteogenic differentiation of MSCs seeded on 3D scaffolds in a process resembling intramembranous ossification (Meijer et al., 2007; Sheehy et al., 2019). However, these strategies have been found to have their own limitations, primarily core degradation, due to a lack of a functional vascular supply upon implantation (Ko et al., 2007; Phelps and Garcia, 2009; O’Brien, 2011; Amini et al., 2012), whereby the formation of a calcified matrix during *in vitro* culture can inhibit *in vivo* vascularization of the graft by sealing up the pores of a scaffold (Lyons et al., 2010; Sheehy et al., 2019). Recent tissue engineering strategies have sought to replicate certain aspects of the endochondral ossification process as it may overcome some of the limitations associated with the traditional intramembranous approach (Jukes et al., 2008; Farrell et al., 2009, 2011; Scotti et al., 2010, 2013; Miot et al., 2012; Freeman et al., 2013, 2015a,b; Harada et al., 2014; Martin, 2014; Gawlitta et al., 2015; Sheehy et al., 2015; Visser et al., 2015; Thompson et al., 2016; Daly et al., 2018). The results thus far have been promising as bone marrow–derived MSCs cultured chondrogenically *in vitro* have an inherent tendency to become hypertrophic, which is the next step in the endochondral ossification pathway that plays a critical role in promoting the conversion of avascular tissue to vascularized tissue, a process that is imperative for the survival of the tissue engineered construct (Farrell et al., 2011; Sheehy et al., 2015, 2019). However, although it has been shown that cartilage templates can become vascularized *in vivo* (Scotti et al., 2013; Daly et al., 2016; Thompson et al., 2016), vascularization and subsequent mineralization occur predominately in the peripheral regions of large tissue engineering constructs whereas avascular cartilage persists at the core (Mesallati et al., 2015; Sheehy et al., 2015; Daly et al., 2018). Previously, we have shown the benefits of both endochondral priming and pre-vascularization of MSC aggregates *in vitro* (Freeman et al., 2015b), as it led to enhanced vessel infiltration into the center of the cellular aggregate when implanted subcutaneously *in vivo* (Freeman et al., 2015a). However, whether this strategy can accelerate and direct vascularization in a scaled-up critical sized defect has yet to be established.

The aim of this study was to directly compare intramembranous and endochondral priming in a critical sized defect by employing a biomaterial delivery construct that supports cell attachment and colonization, and has a highly interconnected porous network to permit tissue in-growth and vascularization when implanted *in vivo* (Navarro et al., 2008; Brennan et al., 2015). Once the optimal priming condition was established, it was then advanced to include endothelialisation prior to implantation, to evaluate the capacity of such tissue engineered implants to accelerate the repair of critically-sized calvaria defects *in vivo*.

## Materials and Methods

### Fabrication of Micro-Fiber PCL Scaffolds

Polycaprolactone (PCL) micro-fiber constructs were supplied by Biomedical Tissues (Nantes, France) and produced as previously described (Abdal-hay et al., 2013; Sohier et al., 2014; Brennan et al., 2015). Briefly, PCL (Sigma Aldrich, molecular weight 80,000 g mol^–1^), was dissolved in chloroform (VWR, Fontenay-sous-Bois, France) to a concentration of 0.1 g mL^–1^ by stirring at 400 rpm at ambient temperature. PCL solution was sprayed using compressed air (8 bars), as the chloroform evaporated a polymer jet was produced, and the micro-fibers were collected on a grid at a distance of 40–50 cm from the spray nozzle. PCL non-woven membranes with a thickness of 400 μm were fabricated and sterilized by gamma irradiation.

### Cell Culture

Bone marrow was collected from the iliac crest as described previously (Brennan et al., 2014), by standard puncture and aspiration into heparinized syringes, from three young, healthy human donors after receiving informed consent according to the Declaration of Helsinki and approval by the Ethical Committee of Ulm University. Human bone marrow stem cells (MSCs) were isolated *ex vivo* by plastic adherence and expanded *in vitro* in triple layered cell stack flasks in standard basal media [αMEM supplemented with 100 U/mL penicillin and 100 μg/mL streptomycin, 10% foetal bovine serum (FBS)]. Human umbilical vein endothelial cells (HUVECs) from one donor were purchased from PromoCell, Heidelberg Germany and cultured in endothelial growth media (EGM-2) (C-22216 basal media with the addition of C-39211 growth medium 2 supplement pack, Promocell). Media were replaced every 3 days and, upon reaching 80–90% confluency, cells were passaged using trypsin-EDTA solution. HUVECs were further cultured to passage 4. For all cell culture performed in this study, cell culture media was changed twice weekly.

### *In vitro* Human Bone Marrow MSC Culture in PCL Micro-Fiber 3D Scaffolds

Mesenchymal stromal cells were seeded onto micro-fiber PCL jet-sprayed scaffolds at a density of 2.7 × 10^4^/cm^2^ and cultured in basal media for up to 21 days. Samples were fixed in 4% paraformaldehyde and rinsed in Phosphate Buffered Saline (PBS). Scanning Electroscope Microscopy (SEM) was used to analyze cell attachment and morphology of MSCs 1.5 h after initial cell seeding. Fixed samples were dehydrated in graded series of ethanol and were mounted on aluminum stubs, sputter coated with gold, and observed with a scanning electron microscope (SEM, Hitachi TM3000, Tokyo, Japan) operating at an acceleration voltage of 5 kV. Cytoskeleton morphology was assessed by fluorescent staining 24 h and 4 days post-seeding. After fixing cells, they were permeabilized with 0.1% Triton X-100 and 0.2% Tween in PBS for 15 min at 4°C followed by incubation with 1% BSA and 5% goat serum at 37°C to reduce non-specific staining. The actin cytoskeleton of MSCs was stained with rhodamine phalloidin (Alexa Fluor 488 Phalloidin, Invitrogen by Life Technologies, Saint Aubin, France) at a dilution of 1/40 with 1% BSA in PBS. Cell nuclei were stained with 4′,6-Diamidino-2-Phenylindole, Dihydrochloride (DAPI, Molecular Probes by Life Technologies) at a concentration of 1/40 000. Images were captured using a Nikon A1R confocal laser-scanning microscope (Nikon, Amstelveen, Netherlands). After 21 days of culture samples were embedded in cryomatrix (Neg 50, Thermoscientific) and submerged in isopentane that was cooled in liquid nitrogen. Cryosections (10 μm thick) were prepared using a cryostat (Micron HM560, Micron Microtech, France). To assess cellular infiltration, frozen sections were air-dried, and fixed in 70% ethanol. Cryosections were processed either by nuclear staining with DAPI and analyzed using fluorescent microscopy (Leica DFC 300 FX), Hematoxylin and Eosin (H&E) staining, or picro-sirus red (All Sigma Aldrich) staining for collagen.

### MSCs Priming in PCL Micro-Fiber Scaffolds Prior to *in vivo* Implantation

A total of 1.25 × 10^5^ MSCs in passages 3–5 were seeded onto the top of PCL scaffolds (8 mm diameter disks) in 20 μL in basal media and incubated for 1 h to allow for cellular attachment, while 10 μL of basal media was added at constant intervals to avoid the scaffold from drying out. After 1 h of incubation the cell seeding procedure was repeated on the opposite side, such that the overall seeding density of the scaffolds was 2.5 × 10^5^ cells/scaffold, comparable to those used previously (Freeman et al., 2015a, b). The seeded scaffolds were cultured for 24 h in basal media, after which they were cultured under the following culture conditions in normoxia: *Unprimed* - cultured in basal media for 21 days; *Endochondral Priming* – cultured in chondrogenic media (chemically defined media which consisted of high-glucose DMEM GlutaMAXTM (Gibco, Life Sciences), 10 ng/ml TGF-β3 (ProSpec-Tany TechnoGene Ltd., Ness-Ziona, Israel), 50 μg/ml ascorbic acid (Sigma-Aldrich), 4.7 μg/ml linoleic acid (Sigma-Aldrich), 100 nM dexamethasone (Sigma-Aldrich) and 1 × insulin–transferrin–selenium (ITS; BD Biosciences, Bedford, MA, United States) for 21 days; *Intramembranous Priming* – cultured in osteogenic media (basal media supplemented with 250 μM ascorbic acid, 10 mM β-glycerolphosphate, and 100 nM dexamethasone) for 21 days. To establish if pre-vascularizing the scaffold prior to implantation would accelerate the *in vivo* angiogenesis and bone healing potential of the endochondrally primed scaffolds the following culture conditions were also performed: *Endochondral Priming* + *HUVECs* – MSC-seeded scaffolds were cultured in chondrogenic media for 21 days after which HUVECs were then seeded on to the scaffolds (125,000 HUVECs/scaffold), using the same process as described above, and cultured for a further 21 days in endothelial growth media prior to implantation; *Endochondral Priming* + *Co-culture –* MSC seeded scaffolds were cultured in chondrogenic media for 21 days after which a 1:1 co-culture of MSCs:HUVECs were then seeded on to the scaffolds (125,000 cells/scaffold), further cultured for another 21 days in endothelial growth media prior to implantation. MSCs from three different human donors were used, with two scaffolds of each priming group were prepared per donor (*n* = 6 scaffolds per priming group).

### Implantation of Micro-Fiber PCL Scaffolds in Calvaria Defects

All animal experiments were performed according to Directive 2010/63/UE and after approval of protocols from the local ethical committee (CEEA, Pays-de-la-Loire, France). Immunocompromised female mice (RjOrl: NMRIFoxn1nu/Foxn1nu) were sourced from a professional breeder (Janvier Labs, Saint-Berthevin, France) at 4 weeks of age. Mice were placed in HEPA filtered cages with water and food *ad libitum* and were quarantined for a minimum of 10 days before surgery. For calvaria implants, the mouse was maintained on a stereostatic frame and a skin incision of 1 cm was made to expose the skull. A 4 mm diameter critical-sized defect was created in the calvaria bone using a trephine and a dental micromotor (Nouvag NM3000; NOUVAG, Goldach, Switzerland). Constant saline irrigation was used during drilling. The cell-laden scaffolds were placed on top of the calvaria defect. Blank scaffolds for each priming condition were incubated for 21 days prior to implantation to serve as controls. Skin incisions were closed with sutures (Filapeau; Peters Surgical, Bobigny, Ile-de-France, France) and analgesic (20 μg/kg; Buprenorphine, Axience, France) was injected intramuscularly before surgery and every 8 h for 3 days after surgery. Animals were observed daily and body weights were determined weekly. After 8 weeks, the mice were euthanized by inhalation of an overdose of carbon dioxide gas. Sample sizes for calvaria implantations were as follows: *blank scaffolds* (basal media, chondrogenic media, osteogenic media, *n* = 2/group); *Unprimed* (Donor 1, *n* = 1, Donor 2, *n* = 2 Donor 3, *n* = 2); *Endochondral Priming* (Donor 1, *n* = 2, Donor 2, *n* = 2 Donor 3, *n* = 2); *Intramembranous Priming* (Donor 1, *n* = 2, Donor 2, *n* = 2 Donor 3, *n* = 2); *Endochondral Priming* + *HUVECs* (Donor 1, *n* = 2, Donor 2, *n* = 2 Donor 3, *n* = 1); *Endochondral Priming* + *Co-culture* (Donor 1, *n* = 2, Donor 2, *n* = 2 Donor 3, *n* = 2).

### X-Ray, Histological, and Immunohistochemical Analysis

Explants were observed for signs of tissue necrosis, inflammation or infection, dissected and fixed in 10 volumes of buffered 4% formaldehyde for 72 h. Using the scoring system previously established (Patel et al., 2008), blind scoring for each planar radiograph (Faxitron MD20, Hologic, United States) was conducted by six impartial people (*n* = 6 scores) to establish the extent of bony bridging and union of the experimental groups.

The skulls were further dissected using a diamond saw. Explants were decalcified in 4.13% ethylenediamine tetraacetic acid (EDTA)/0.2% paraformaldehyde in phosphate-buffered saline, pH 7.4 for 96 h at 50°C using an automated microwave decalcifying apparatus (KOS Histostation; Milestone Medical, Kalamazoo, Michigan, United States). Samples were then dehydrated in ascending series of ethanol baths (80, 95, and 100%) and finally in butanol in an automated dehydration station (Microm Microtech, Lyon, France), and then embedded in paraffin (Histowax; Histolab, Gottenburg, Sweden). Blocks were cut using a standard microtome (Leica RM2255; Leica Biosystems, Nanterre, Ile-de-France, France) and histology sections (5–8 μm thick) in the middle of calvaria defects were made. Sections were stained by Masson trichrome technique using an automated coloration station (Microm Microtech). Histomorphometry of images were processed on the whole implant sections using Image J software (National Institute of Health, Bethesda, MA, United States) and the percentage areas of bone tissue per total area of the calvaria defect was measured. Sections were also stained with Hematoxylin and Eosin (H&E, Sigma Aldrich) and Goldner’s Trichrome (Hematoxyline de Groat, Fuchsine Ponceau, 0.1% Orange G molybdique, 2% Fast Green, All Sigma Aldrich) and quantified for vessel infiltration, whereby vessels (positive staining for endothelium and erythrocytes present within the lumen), were counted on separate sections (*n* = 3 slices per defect) and a taken throughout each construct.

To identify the specific collagen types, immunohistochemistry was performed for collagen type I and II, as previously described (Buckley et al., 2010; Browe et al., 2019). Briefly, after dewaxing and rehydrating the sections antigen retrieval was performed by incubation with Chondrotinase ABC for collagen types I and II. After blocking for non-specific binding, sections were incubated with primary antibody (anti collagen type I (1:400), Abcam, United Kingdom; anti collagen type II (1:400), Santa-Cruz) overnight at 4°C. Endogenous peroxidase activity was blocked with hydrogen peroxide (Sigma) prior to incubation with the anti-mouse IgG secondary antibody (Sigma). Sections were then incubated with 3,30 -diaminobenzidine peroxidase substrate (Vector Labs, United Kingdom) to visualize positive staining. All Stained slices were scanned (NanoZoomer; Hamamatsu, Photonics, Hamamatsu City, Shizuoka Prefecture, Japan) and observed on a virtual microscope (NDP view; Hamamatsu).

### Statistical Analysis

Results were expressed as mean ± standard deviation. Statistical analysis was performed using one-way analyses of variance (ANOVA) with the addition of Tukey’s correction for multiple comparisons testing. All analyses were performed using GraphPad (GraphPad Software, La Jolla, CA, United States)^1^. For all comparisons, the level of significance was *p* ≤ 0.05.

## Results

### PCL Micro-Fiber Scaffold Permitted MSC Spreading, Infiltration and Matrix Formation

**FIGURE 1:**
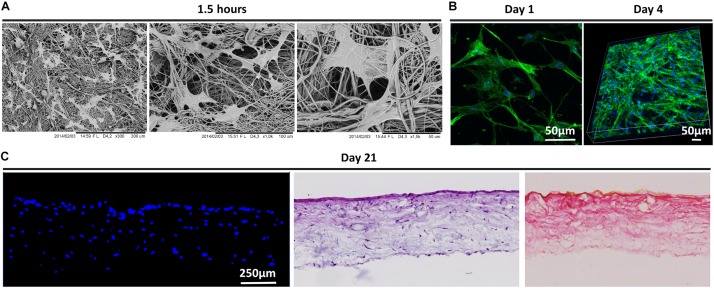
**(A)** Morphology of human bone marrow MSCs attached to scaffolds 1.5 h after cell seeding as observed by SEM. Black scale bars represent 300 and 100 μm on the left and right, respectively. **(B)** Confocal microscopy of MSCs on scaffolds 24 h and 4 days after seeding cells. Actin cytoskeleton arrangement is shown in green by fluorescent staining with rhodamine phalloidin and nuclei are depicted in blue by DAPI staining. White scale bars represent 50 μm. **(C)** MSCs infiltration into scaffolds after 21 days post-culture as shown by fluorescent DAPI staining of cell nuclei in scaffold cryo-sections and H&E staining. Collagen matrix formation is observed in pink by pico-sirus red staining. Scale bars represents 250 μm.

The morphology of MSCs following attachment to PCL scaffolds was visualized using SEM and confocal imaging of fluorescently stained cells (Figures 1A,B). As early as 1.5 h after seeding, MSCs were well attached to the scaffold along the lengths of the scaffold micro-fibers and exhibit an elongated morphology (Figure 1B). Confocal imaging after 1 and 4 days shows that MSCs assumed a spread morphology and were orientated in different directions along the struts of the scaffolds, with intense cytoskeleton staining. Cell ingress into the scaffolds was observed by DAPI stained cell nuclei and H&E staining of scaffold cross sections. As demonstrated in Figure 1C, by day 21 MSCs penetrated through the entire depth of the scaffolds and exhibited significant collagen matrix deposition as shown by the pink staining (pico sirus red).

### Endochondral Priming of the Scaffolds Enhanced Vessel Infiltration and Lead to Increased Bone Union

**FIGURE 2:**
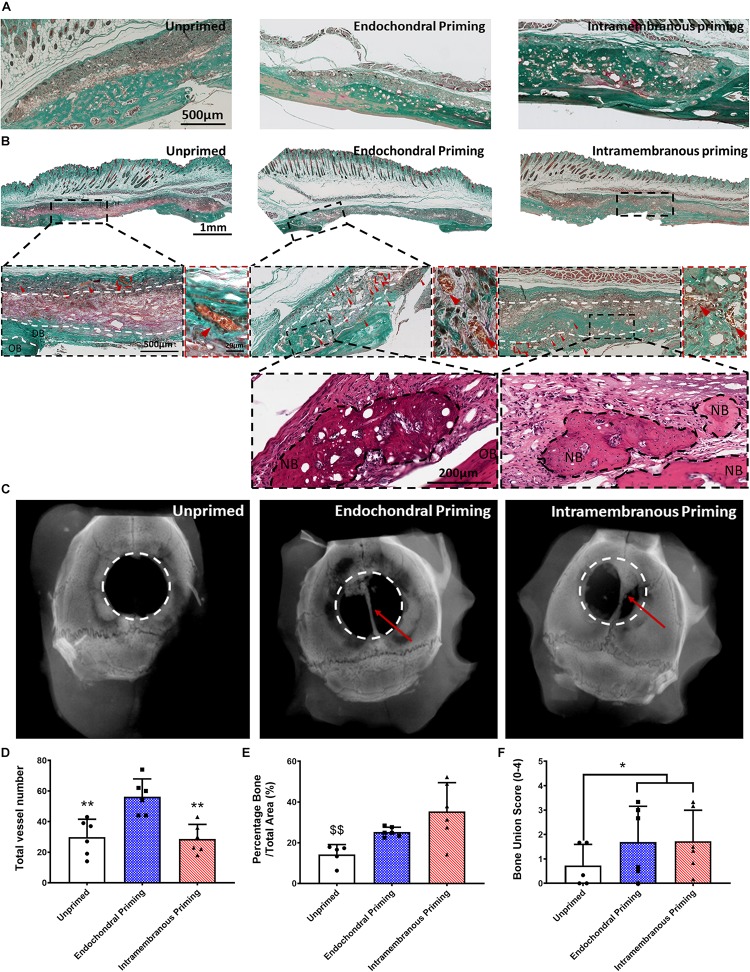
**(A)** Masson’s Trichrome stained sections of all groups after 8 weeks *in vivo*, showing the osteoconductive nature of the scaffolds. **(B)** Masson’s Trichrome and H&E stained sections of all groups taken in the middle of the defect after 8 weeks *in vivo.* All Images taken at 20X. White dashed lines denoting periphery and center, OB denoted original bone, and NB denotes new bone. Red arrow heads denote vessels. **(C)** Representative X-ray images of the three experimental groups 8 weeks after implantation. Quantification of the amount of panel **(D)** total number of vessels, **(E)** percentage new bone, and **(F)** bone union score for all three experimental groups 8 weeks post implantation. Error bars denote standard deviation, **p < 0.01 vs. Endochondral Priming group, ^$$^*p* < 0.01 vs. Intramembranous Priming group, *n* = 6 animals.

To assess the osteoconductive nature of the scaffolds 8 weeks-post implantation, we looked at areas within the defect where the scaffold was laid upon undamaged calvaria bone (Figure 2A). Goldner’s Trichrome staining revealed abundant bone formation within the scaffolds and that there did not seem to be any differences in the osteoconductive nature of the scaffolds between all three groups, elucidating that the osteoconductivity was due to the designed PCL scaffold and not the culture conditions. When examined in the center of the defects, all defects treated with the controlled blank scaffolds were filled with fibrous tissue, as seen in the positive red staining (see Supplementary Figure S1A). They also all showed limited vessel infiltration, new bone formation and bone union (see Supplementary Figures S1B–E). Masson’s Trichrome staining revealed predominantly fibrous tissue formation, similar to what was seen in the blank scaffolds, in the *Unprimed* group (Figure 2B). On the other hand, in the defects of the *Endochondral* and *Intramembranous Primed* groups there was little to no fibrous tissue present. Histological analysis of H&E and Goldner’s Trichrome stained samples revealed the presence of vessels in all three experimental groups (denoted by red arrow heads). These vessels appeared mature with endothelium and perfused with erythrocytes (see Supplementary Figure S2). The *Unprimed* and *Intramembranous Primed* groups had vessels predominantly located in the periphery of the scaffold, with little to none present within the center of the scaffold (denoted by the white dashed lines). In contrast, vessels were present both in the periphery and in the center of the *Endochondral Primed* group. When quantified there was significantly more vessels (*p* < 0.01) present in the *Endochondral Primed* group compared to both the *Intramembranous* and *Unprimed* groups (Figure 2D). Next, we sought to assess bone regeneration capability of the scaffolds under the different priming conditions. First, Masson’s Trichrome and H&E staining revealed there was positive staining for new bone, complete with marrow cavities, in both the *Intramembranous* and *Endochondral Primed* groups 8 weeks post-implantation (see Figure 2B). When quantified, there was significantly more new bone (*p* < 0.01) found in the *Intramembranous Primed* group compared to the *Unprimed* group (see Figure 2E). This was further verified using immunohistochemistry where the *Intramembranous Primed* group had the highest amount of positive Collagen Type I staining whereas the *Endochondral Primed* group had the highest amount of positive Collagen Type II staining (Figure 4A). There was no significant difference in percentage new bone formed between the *Intramembranous* and the *Endochondral Primed* groups. Although the X-ray analysis revealed limited bone healing in all three groups, the *Unprimed* group had the poorest healing potential, with no bone unions present in any of the animals within this group (see Figure 2C). Interestingly, in the other two treatment groups there was clear healing along the sagittal suture line of the mice craniums (denoted by red arrows). In fact, 33 and 50% of the animals had full bone union in the *Intramembranous* and *Endochondral Primed* groups respectively. When scored blind, the *Endochondral* and *Intramembranous Primed* groups had significantly higher (*p* < 0.05) bone union score than the *Unprimed* group (Figure 2F).

### Pre-vascularization of the Cartilage Template did Not Further Enhance the Bone Healing Potential of the Scaffolds

**FIGURE 3:**
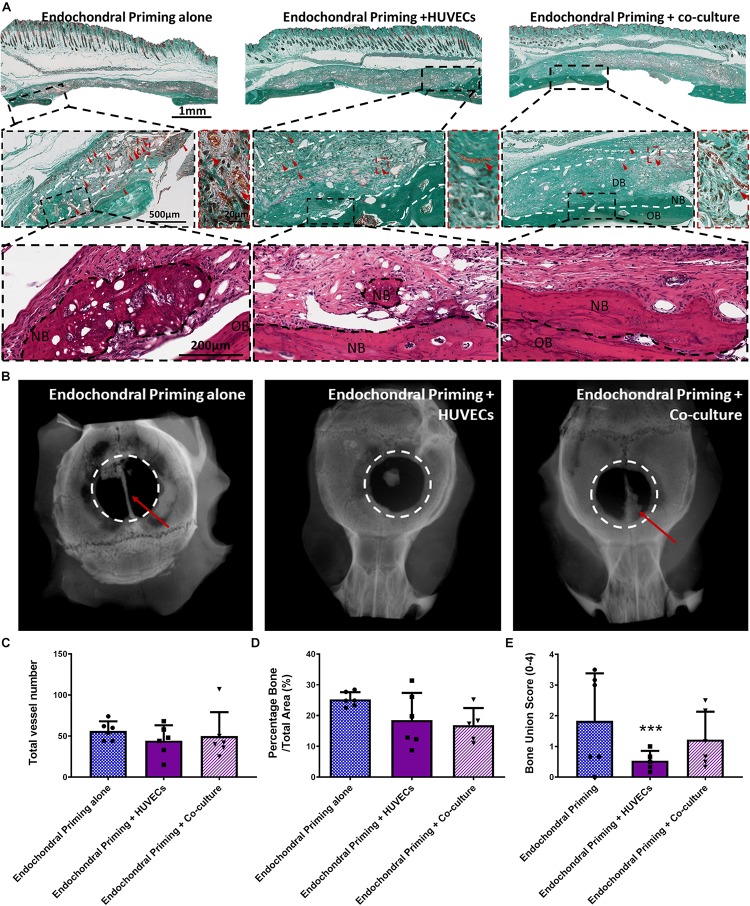
**(A)** Masson’s Trichrome and H&E stained sections of all groups taken in the middle of the defect after 8 weeks *in vivo.* All Images taken at 20X. White dashed lines denoting periphery and centre, OB denoted original bone, and NB denotes new bone. Red arrow heads denote vessels. **(B)** Representative X-ray images of the three experimental groups 8 weeks after implantation. Quantification of the amount of panel **(C)** total number of vessels, **(D)** percentage new bone, and **(E)** bone union score for all three experimental groups 8 weeks post implantation. Error bars denote standard deviation, ***p < 0.01 vs. Endochondral Priming alone group, *n* = 6 animals.

**FIGURE 4:**
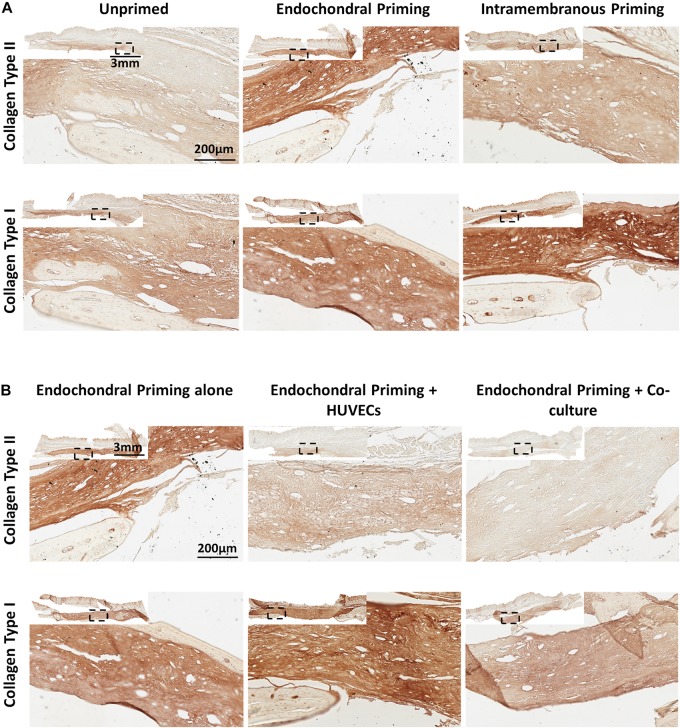
**(A)** Immunohistochemistry (Collage Type I and Collagen Type II) stained sections of *Unprimed, Endochondral and Intramembranous primed* groups taken in the middle of the defect after 8 weeks *in vivo.* All Images taken at 20X. **(B)** Immunohistochemistry (Collage Type I and Collagen Type II) stained sections of *Endochondral priming alone, Endochondral priming* + *HUVECs and Endochondral priming* + *Co-culture* groups taken in the middle of the defect after 8 weeks *in vivo.* All Images taken at 20X.

Having demonstrated the potential of endochondral priming but recognizing that only limited healing was achieved after 8 weeks, we next sought to examine if we could further accelerate the bone healing capacity of the constructs by pre-vascularizing them *in vitro* prior to implantation. There was no sign of fibrous tissue formation in any of the defects treated with all three experimental groups (Figure 3A). All three groups had vessels present throughout the defects, and when quantified there was no significant difference in vessel number between any of the groups (Figure 3C). We next sought to assess the nature of new bone tissue being formed using histological staining. All three experimental groups had positive staining for new bone and when quantified there was no increase in new bone formed due to the pre-vascularization process (Figure 3D). This was further verified as all three groups had positive staining for Collagen Type I (Figure 4B). Interestingly, the X-rays reveal a difference in where the bone was formed. In both the *Endochondral Primed* and the *Endochondral Primed* + *Co-culture* groups, similar to what was seen previously, bone healed along the sagittal suture line (Figure 3B). However, in the *Endochondral Primed* + *HUVECs* group bone was formed sporadically with a few bony spicules dispersed throughout the defect. In fact, all the *Endochondral Primed* + *HUVECs* group were non-union defects after 8 weeks. Whereas, 50 and 17% of the animals had full bone bridging in the *Endochondral Primed* and the *Endochondral Primed* + *Co-culture* groups, respectively. When scored blind the *Endochondral Primed* group had a significantly higher (*p* < 0.001) bone union score than the *Endochondral Primed* + *HUVECs* group (Figure 3E). There was no significant difference between the *Endochondral Primed* group and the *Endochondral Primed* + *Co-culture* group.

## Discussion

To date most bone tissue engineering strategies that have reached the clinic have tried to produce a construct that mimics the function or mechanical properties of native bone tissue, and although this strategy has produced extensive amount of research, *in vitro* tissue regeneration constructs for the clinical treatment of bone defects has not reached its full potential (Frohlich et al., 2008; Ma et al., 2014). In this vein, recent tissue engineering strategies have sought to replicate features that occur during embryogenesis or “developmental engineering” (Jukes et al., 2008; Farrell et al., 2009, 2011; Lenas et al., 2009; Scotti et al., 2010, 2013; Miot et al., 2012; Freeman et al., 2013, 2015a,b; Harada et al., 2014; Martin, 2014; Gawlitta et al., 2015; Sheehy et al., 2015; Visser et al., 2015; Thompson et al., 2016; Daly et al., 2018). The results from this study further demonstrate the tremendous potential of “developmental engineering,” as both intramembranous and endochondral priming showing a significant increase in new bone formation over scaffolds with MSCs that were not developmentally primed. Furthermore, endochondral priming alone was sufficient to increase bone healing, but further endothelialisation provided no benefit or acceleration in vessel infiltration or bone healing in a critical-sized defect.

Essential features of biomaterials for bone reconstruction include; a structure that supports osteogenic cell responses, appropriate biodegradability and biocompatibility, and a highly interconnected porous network to permit tissue in-growth and vascularization (Navarro et al., 2008). There are currently several commercial products which utilize collagen to direct bone repair. However, in order to avoid rapid degradation, collagen is usually cross-linked, and the use of chemical cross-linkers such as glutaraldehyde can cause long-term cytotoxicity (van Wachem et al., 1994; Marinucci et al., 2003). Cross-linking also reduced scaffold pore sizes which have been shown to inhibit vascularization (Shields et al., 2004). In this study, instead of recapitulating the native collagen ECM, a biomimetic polymer biomaterial was developed to mimic the nanofibrous structure of the ECM. This scaffold not only allowed for cell infiltration but also promoted spread morphology with intense actin cytoskeleton staining. It has previously been shown that MSCs with a large spreading area showed a higher degree of osteogenic differentiation (Yang et al., 2019) and indeed, we previously showed that this micro-fiber scaffold with highly interconnected porous network promoted osteogenic cell responses *in vitro* (Brennan et al., 2015). Here we show that the microfiber PCL scaffold permits tissue in-growth, vascularization and also supports osteogenesis *in vivo*.

We next sought to investigate the bone healing potential of MSC-laden PCL scaffolds that were first primed for either intramembranous or endochondral prior to implantation. During early fotal development the cranium is formed via the intramembranous ossification process and in this study, we investigated the optimum priming condition to enhance the regeneration potential of a calvaria critical sized defect model. The bone healing potential was significantly increased if the scaffolds where loaded with MSCs that were first primed along either an intramembranous or endochondral pathway. Intramembranous bone growth is achieved through bone formation within a periosteum or by bone formation at suture lines (Opperman, 2000). Interestingly, the bone healing pattern seen in this study was characterized by new bone predominately laid down along the sagittal suture line of the cranium. Interestingly, endochondral priming of the cells prior to implantation does not change this bone formation pattern, with 50% of the animals (vs. 33% for intermembranous) having full bone bridging along the sagittal suture line, there was a trend toward increased bone regeneration potential. Directly comparing the bone formation of the two priming conditions, there was no significant difference in the bone healing capacity between either group. Similar to previous studies (Thompson et al., 2016), histomorphological analysis showed an increase in percentage new bone in the intramembranous group over the endochondral ossification group, however the trend was not significant. Furthermore, similar to previous studies (Thompson et al., 2016), there was significantly more vessels present in the endochondral primed constructs over the intramembranous primed constructs. Unlike previous studies (Mesallati et al., 2015; Sheehy et al., 2015; Thompson et al., 2016; Daly et al., 2018), the vascularization was throughout the defect and not predominately in the peripheral regions of large tissue engineering constructs. It was due to this significant increase in vessel infiltration that endochondral priming was chosen as the optimum priming condition and was taken forward to be endothelialised prior to implantation. Exogenous osteogenic and chondrogenic growth factors (ascorbic acid, dexamethasone, β-glycerol, and TGF-β3) were introduced into the culture media of MSCs to encourage MSC differentiation down to specific pathways. Therefore, in order to clearly distinguish whether any of these factors contributed to the differences seen between the groups we includeed control PCL scaffold groups that were exposed to the same osteogenic or chondrogenic factors, but with no cells, and observed no increase in bone healing or vascularization, leading us to believe that the therapeutic effect in the experimental groups is due to the priming of the MSCs and not due to the presence of the exogenous growth factors.

One potential limitation to the study was that we did not investigate the cell viability of the human MSCs post-implantation. Previously, we have shown that following subcutaneous implantation of endochondrally primed and prevascularised human MSC cellular aggregates, the human MSCs survived up to 21 days (Freeman et al., 2015a). This correlated with other studies which have investigated the cell viability of human MSCs following implantation (Vilalta et al., 2008; Brennan et al., 2014; Manassero et al., 2016). With this in mind, even though the primed human MSCs may not survive the entire implantation their presence starts a cascade of events *in vivo* toward increased bone regeneration. Future work should delve further into understanding the cell viability and the exact role the primed MSCs have on the bone regeneration capacity of the implants.

The addition of endothelial cells prior to implantation did not increase the bone healing or vascularization potential of the endochondral primed constructs. In fact, when endothelial cells alone were added to the endochondral primed construct, it hindered the bone healing capacity of the construct. This was a complete contradiction to our previous work (Freeman et al., 2015a, b), where the mineralization of cellular aggregates was improved through the pre-vascularization process. This could may be explained by the fact that during endochondral ossification, hypertrophic chondrocyte secretes VEGF, which is a potent chemoattractant for the recruitment of endothelial cells and osteoclasts and promotes cartilage resorption. The addition of endothelial cells to hypertrophic chondrocytes present in the endochondral primed construct, may suppress the hypertrophic chondrocytes from secreting VEGF, as endothelial cells are already present, thereby, hindering the bone healing process (Hans-Peter et al., 1999; Gerber and Ferrara, 2000). Interestingly, the healing capacity is restored when both MSCs and endothelial cells are added in a co-culture. This further cooperates with our hypothesis because in this approach half the number of endothelial cells was added to the scaffold, which may have minimized communication between hypertrophic chondrocytes and endothelial cells and healing capacity was restored. However, future studies should look further into the direct communication between the hypertrophic cartilage template and endothelial cells to elucidate this finding further and determine an appropriate methodology for pre-vascularizing an endochondral primed construct. Taken together, the results presented in this study demonstrate that endochondral priming alone is enough to induce vascularization and subsequent bone healing in a critically sized defect.

## Data Availability Statement

The datasets generated for this study are available on request to the corresponding author.

## Ethics Statement

All animal experiments were performed according to Directive 2010/63/UE and after approval of protocols from the local ethical committee (CEEA, Pays-de-la-Loire, France).

## Author Contributions

FF and MB performed the experiments, data analysis and interpretation, and wrote the manuscript. DB performed all the immunohistochemistry. AR performed all the animal surgeries. JD sliced and performed some of the Masson’s Trichrome Histology. DK, LM, and PL oversaw the collection of results and data interpretation and finalized the manuscript.

## Conflict of Interest

Research undertaken in DK’s laboratory at Trinity College Dublin is part-funded by Johnson & Johnson. The funder was not involved in the study design, collection, analysis, interpretation of data, the writing of this article or the decision to submit it for publication. The remaining authors declare that the research was conducted in the absence of any commercial or financial relationships that could be construed as a potential conflict of interest.

## Footnotes

1 www.graphpad.com

## Supplementary Material

FIGURE S1 **(A)** Masson’s Trichrome stained sections of control scaffolds after 8 weeks *in vivo*. Images taken at 20X. **(B)** Representative X-ray images of the three control groups 8 weeks after implantation. Quantification of the amount of panel **(C)** total number of vessels, **(D)** percentage new bone, and **(E)** bone union score for all three control groups 8 weeks post implantation. Error bars denote standard deviation, *n* = 2 animals.

FIGURE S2 Goldner’s Trichrome stained sections of control scaffolds after 8 weeks *in vivo*. Red arrow heads denote mature vessels perfused with erythrocytes. Images taken at 20X.
