# Supplementary figures and images for: A Developmental Engineering-Based Approach to Bone Repair: Endochondral Priming Enhances Vascularization and New Bone Formation in a Critical Size Defect
Source: Front Bioeng Biotechnol. 2020 Mar 31;8:230. doi: 10.3389/fbioe.2020.00230 (PMC7137087; doi:10.3389/fbioe.2020.00230)

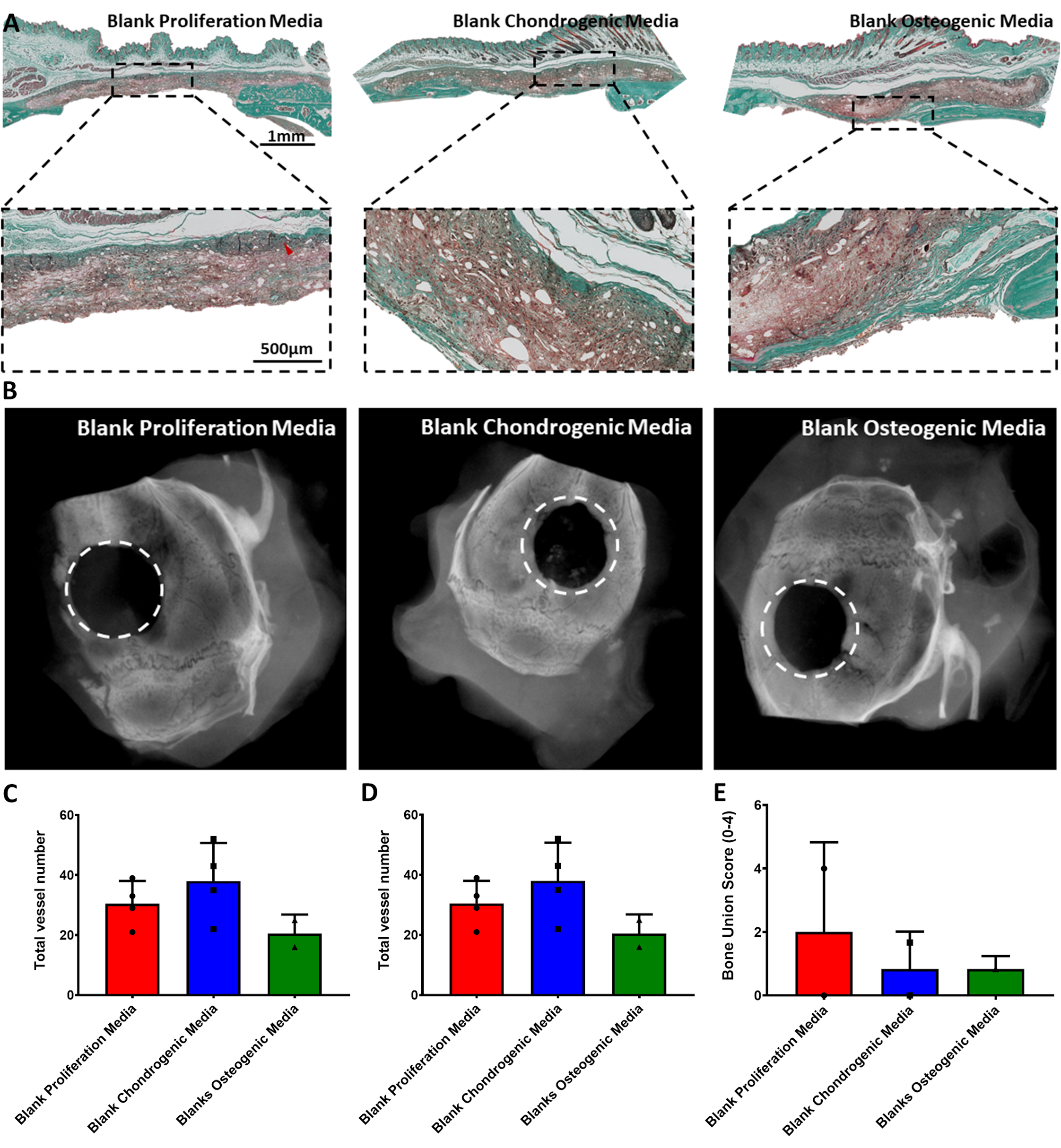

Supplement: FIGURE S1 — (A) Masson’s Trichrome stained sections of control scaffolds after 8 weeks in vivo. Images taken at 20X. (B) Representative X-ray images of the three control groups 8 weeks after implantation. Quantification of the amount of panel (C) total number of vessels, (D) percentage new bone, and (E) bone union score for all three control groups 8 weeks post implantation. Error bars denote standard deviation, n = 2 animals. [file Image_1.TIF]

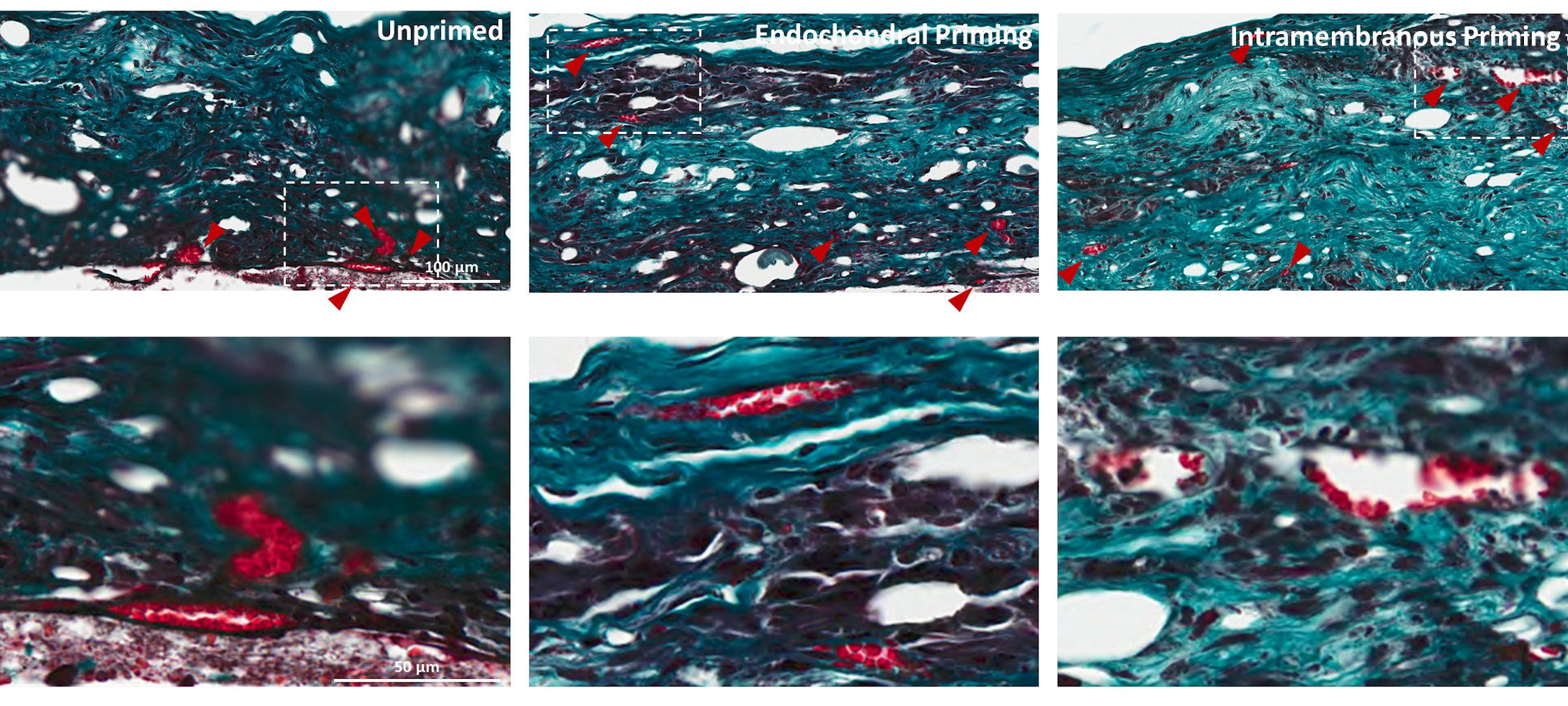

Supplement: FIGURE S2 — Goldner’s Trichrome stained sections of control scaffolds after 8 weeks in vivo. Red arrow heads denote mature vessels perfused with erythrocytes. Images taken at 20X. [file Image_2.TIF]
